# Supplementary material for: Protective Role of IRBIT on Sodium Bicarbonate Cotransporter-n1 for Migratory Cancer Cells
Source: Pharmaceutics. 2020 Aug 27;12(9):816. doi: 10.3390/pharmaceutics12090816 (PMC7558343; doi:10.3390/pharmaceutics12090816)
Supplement: Supplementary file 1 [file pharmaceutics-12-00816-s001.zip › pharmaceutics-881487-supplementary.pptx]

## Slide 1
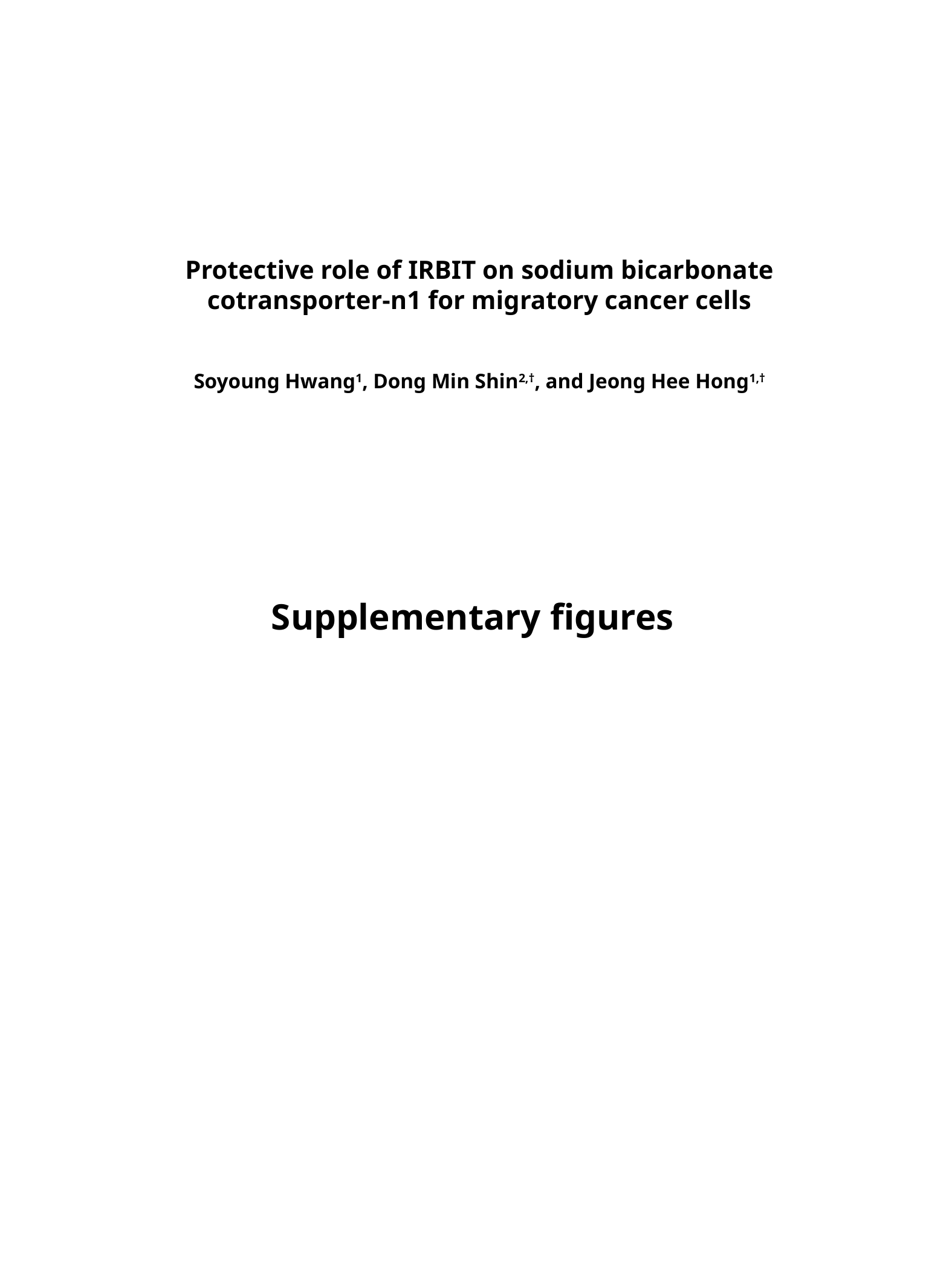

Protective role of IRBIT on sodium bicarbonate cotransporter-n1 for migratory cancer cells
Soyoung Hwang1, Dong Min Shin2,†, and Jeong Hee Hong1,†
# Supplementary figures

## Slide 2
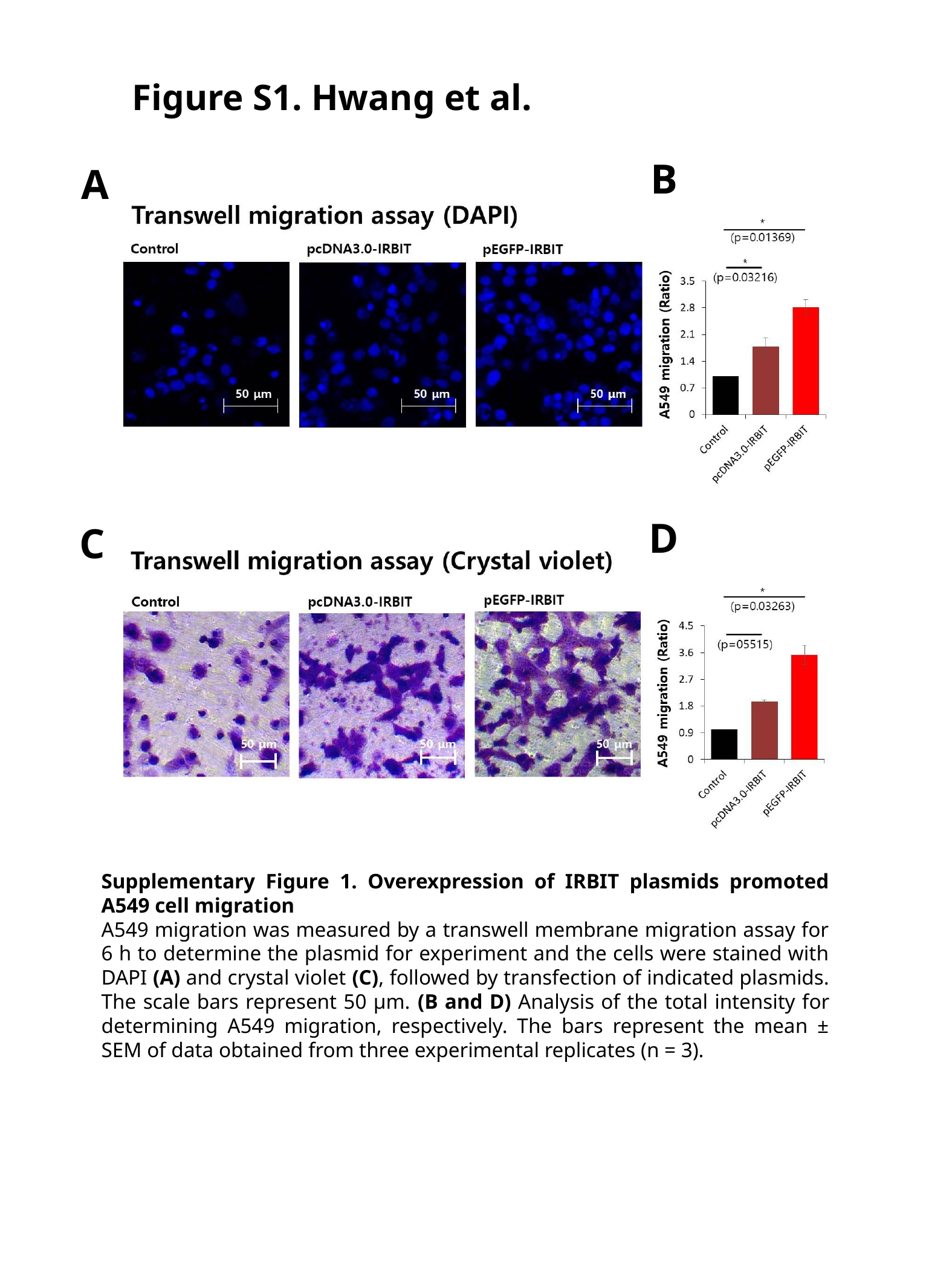

Figure S1. Hwang et al.
B
A
D
C
Supplementary Figure 1. Overexpression of IRBIT plasmids promoted A549 cell migration
A549 migration was measured by a transwell membrane migration assay for 6 h to determine the plasmid for experiment and the cells were stained with DAPI (A) and crystal violet (C), followed by transfection of indicated plasmids. The scale bars represent 50 μm. (B and D) Analysis of the total intensity for determining A549 migration, respectively. The bars represent the mean ± SEM of data obtained from three experimental replicates (n = 3).

## Slide 3
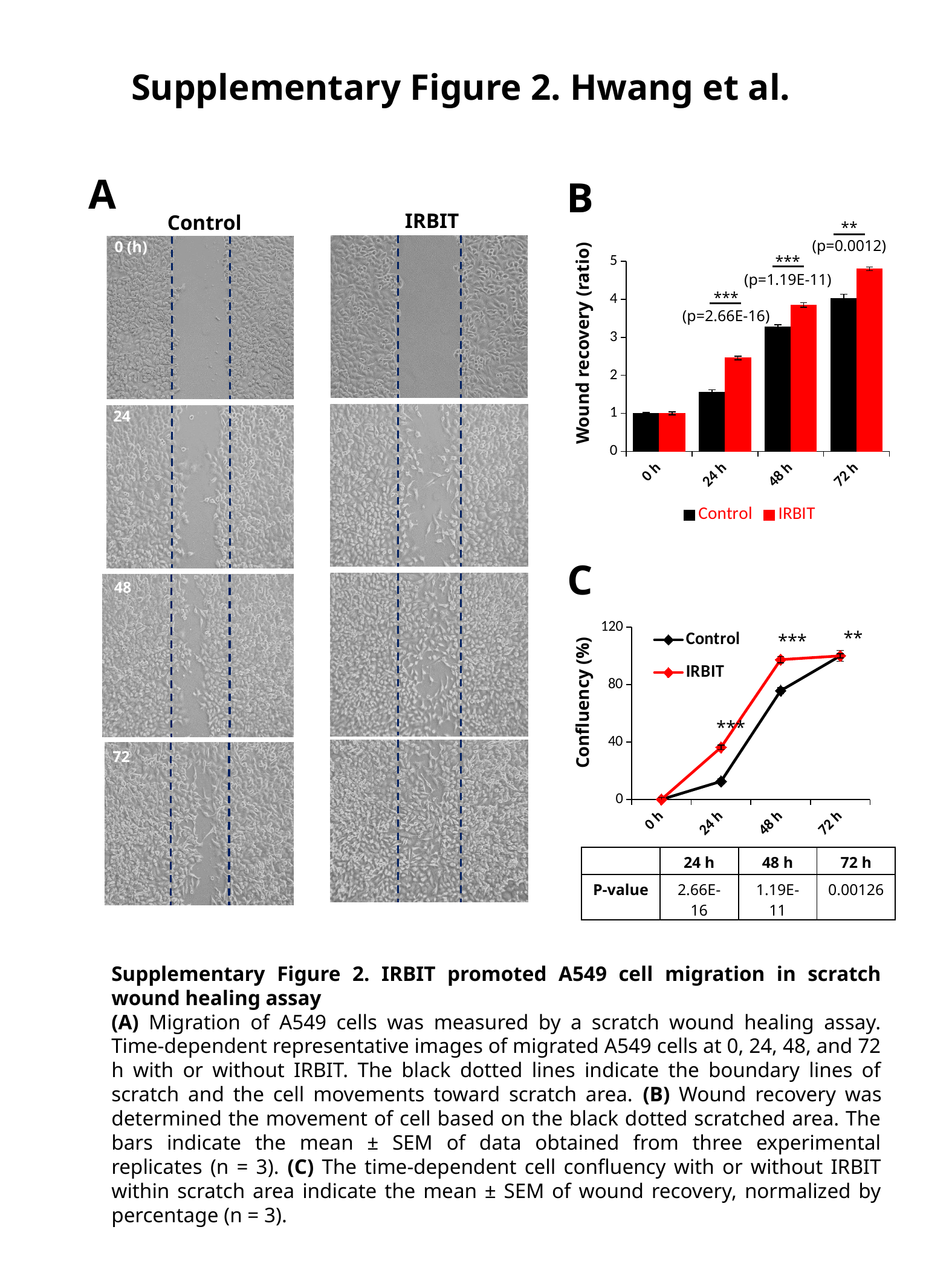

Supplementary Figure 2. Hwang et al.
A
B
Wound recovery (ratio)
IRBIT
Control
**
(p=0.0012)
0 (h)
***
(p=1.19E-11)
### Chart
| Category | Control | IRBIT |
|---|---|---|
| 0 h | 1.0 | 1.0 |
| 24 h | 1.558947190687603 | 2.4605023978960388 |
| 48 h | 3.2727677959536363 | 3.854957690536494 |
| 72 h | 4.02267692611842 | 4.806755537841919 |***
(p=2.66E-16)
24
C
48
### Chart
| Category | Control | IRBIT |
|---|---|---|
| 0 h | 0.0 | 0.0 |
| 24 h | 12.5996 | 36.29722 |
| 48 h | 75.74099 | 97.3838 |
| 72 h | 100.0 | 100.0 |**
***
 Confluency (%)
***
72
| | 24 h | 48 h | 72 h |
| --- | --- | --- | --- |
| P-value | 2.66E-16 | 1.19E-11 | 0.00126 |
Supplementary Figure 2. IRBIT promoted A549 cell migration in scratch wound healing assay
(A) Migration of A549 cells was measured by a scratch wound healing assay. Time-dependent representative images of migrated A549 cells at 0, 24, 48, and 72 h with or without IRBIT. The black dotted lines indicate the boundary lines of scratch and the cell movements toward scratch area. (B) Wound recovery was determined the movement of cell based on the black dotted scratched area. The bars indicate the mean ± SEM of data obtained from three experimental replicates (n = 3). (C) The time-dependent cell confluency with or without IRBIT within scratch area indicate the mean ± SEM of wound recovery, normalized by percentage (n = 3).

## Slide 4
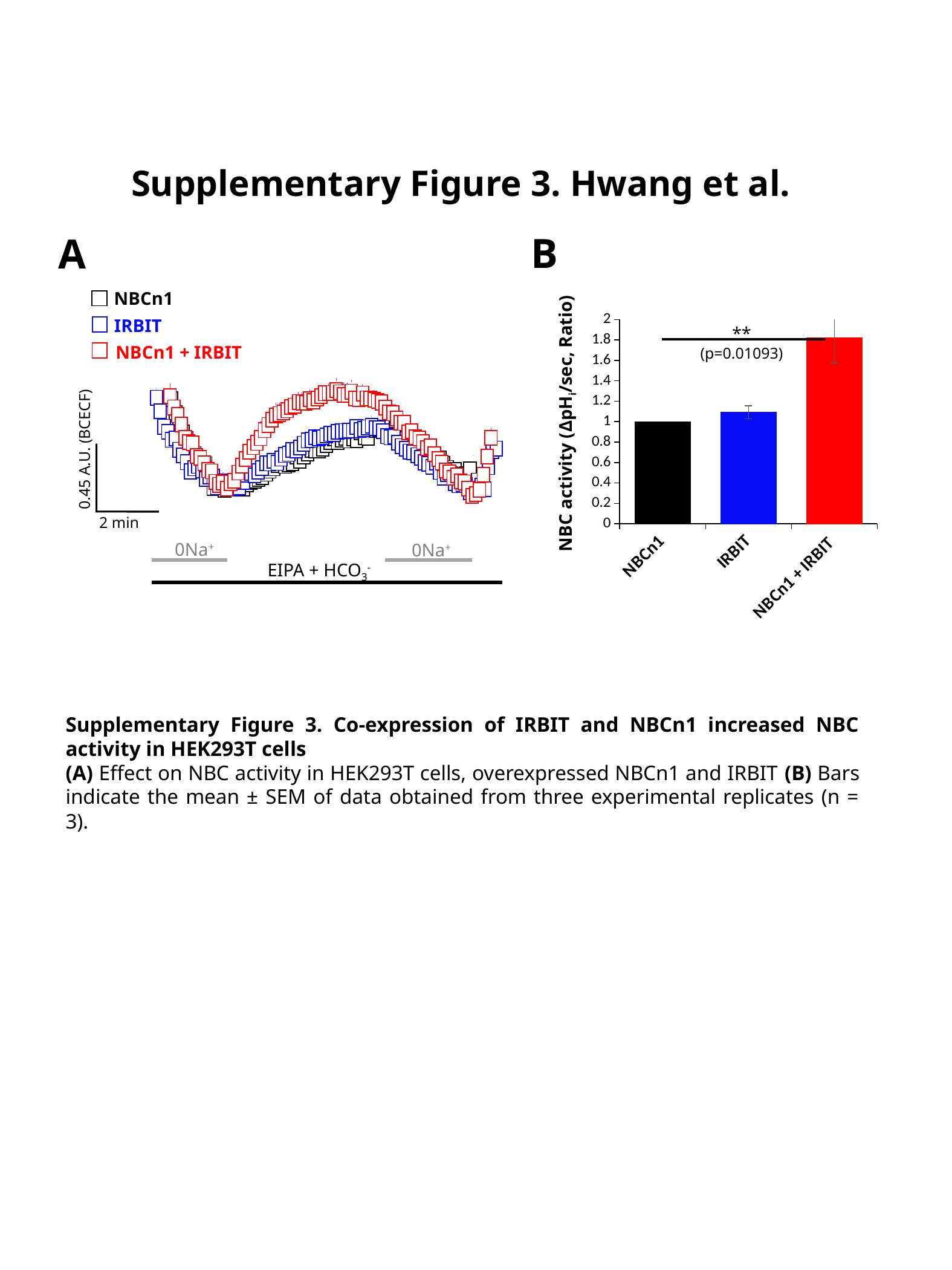

Supplementary Figure 3. Hwang et al.
B
A
 NBCn1
 IRBIT
 NBCn1 + IRBIT
NBC activity (ΔpHi/sec, Ratio)
### Chart
| Category | AV |
|---|---|
| NBCn1 | 1.0 |
| IRBIT | 1.0912263496456762 |
| NBCn1 + IRBIT | 1.8221006636083297 |**
(p=0.01093)
0.45 A.U. (BCECF)
2 min
0Na+
0Na+
EIPA + HCO3-
Supplementary Figure 3. Co-expression of IRBIT and NBCn1 increased NBC activity in HEK293T cells
(A) Effect on NBC activity in HEK293T cells, overexpressed NBCn1 and IRBIT (B) Bars indicate the mean ± SEM of data obtained from three experimental replicates (n = 3).

## Slide 5
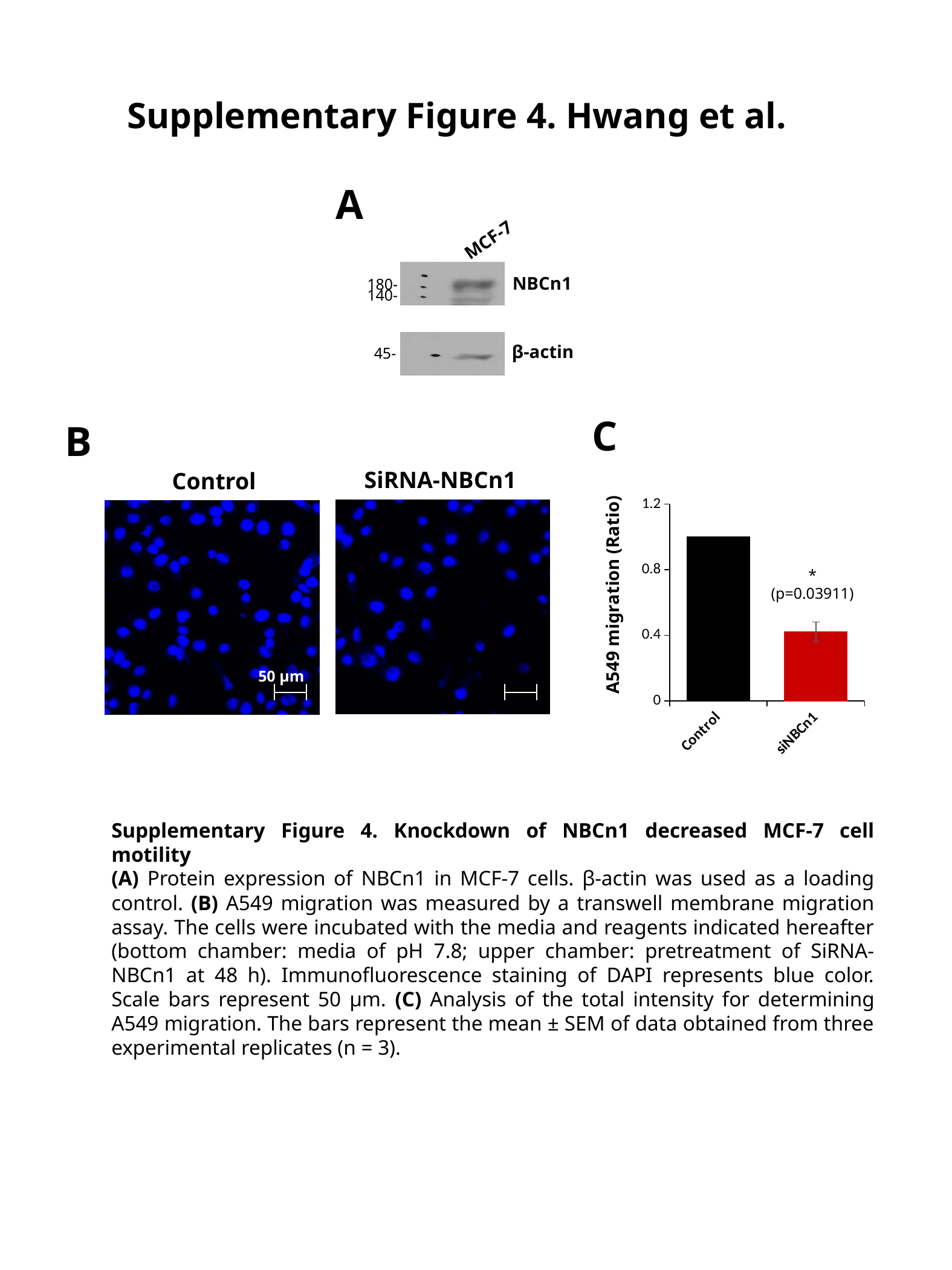

Supplementary Figure 4. Hwang et al.
A
MCF-7
NBCn1
180-
140-
β-actin
 45-
C
B
SiRNA-NBCn1
Control
### Chart
| Category | av |
|---|---|
| Control | 1.0 |
| siNBCn1 | 0.42169130174079394 |A549 migration (Ratio)
*
(p=0.03911)
 50 μm
Supplementary Figure 4. Knockdown of NBCn1 decreased MCF-7 cell motility
(A) Protein expression of NBCn1 in MCF-7 cells. β-actin was used as a loading control. (B) A549 migration was measured by a transwell membrane migration assay. The cells were incubated with the media and reagents indicated hereafter (bottom chamber: media of pH 7.8; upper chamber: pretreatment of SiRNA-NBCn1 at 48 h). Immunofluorescence staining of DAPI represents blue color. Scale bars represent 50 μm. (C) Analysis of the total intensity for determining A549 migration. The bars represent the mean ± SEM of data obtained from three experimental replicates (n = 3).

## Slide 6
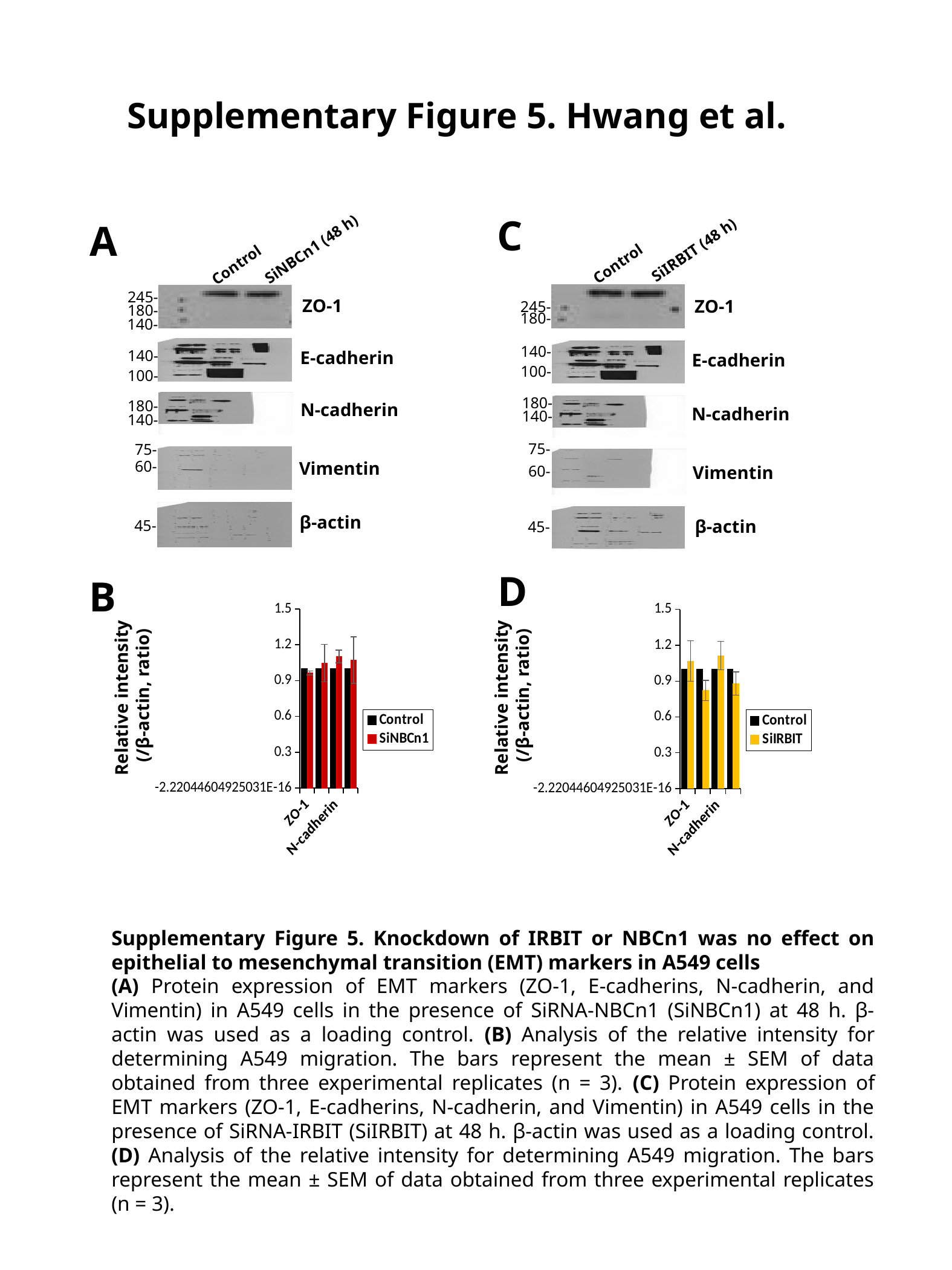

Supplementary Figure 5. Hwang et al.
C
A
SiNBCn1 (48 h)
SiIRBIT (48 h)
Control
Control
245-
ZO-1
ZO-1
245-
180-
180-
140-
140-
100-
E-cadherin
E-cadherin
140-
100-
180-
140-
180-
140-
N-cadherin
N-cadherin
 75-
 75-
 60-
Vimentin
Vimentin
 60-
β-actin
β-actin
 45-
 45-
D
B
### Chart
| Category | Control | SiNBCn1 |
|---|---|---|
| ZO-1 | 1.0 | 0.9643051474277511 |
| E-cadherin | 1.0 | 1.045412073899462 |
| N-cadherin | 1.0 | 1.1018083427625884 |
| Vimentin | 1.0 | 1.071609353425641 |
### Chart
| Category | Control | SiIRBIT |
|---|---|---|
| ZO-1 | 1.0 | 1.0679281170120383 |
| E-cadherin | 1.0 | 0.821111008913133 |
| N-cadherin | 1.0 | 1.1137990998027893 |
| Vimentin | 1.0 | 0.8800603605534395 |Relative intensity
(/β-actin, ratio)
Relative intensity
(/β-actin, ratio)
Supplementary Figure 5. Knockdown of IRBIT or NBCn1 was no effect on epithelial to mesenchymal transition (EMT) markers in A549 cells
(A) Protein expression of EMT markers (ZO-1, E-cadherins, N-cadherin, and Vimentin) in A549 cells in the presence of SiRNA-NBCn1 (SiNBCn1) at 48 h. β-actin was used as a loading control. (B) Analysis of the relative intensity for determining A549 migration. The bars represent the mean ± SEM of data obtained from three experimental replicates (n = 3). (C) Protein expression of EMT markers (ZO-1, E-cadherins, N-cadherin, and Vimentin) in A549 cells in the presence of SiRNA-IRBIT (SiIRBIT) at 48 h. β-actin was used as a loading control. (D) Analysis of the relative intensity for determining A549 migration. The bars represent the mean ± SEM of data obtained from three experimental replicates (n = 3).

## Slide 7
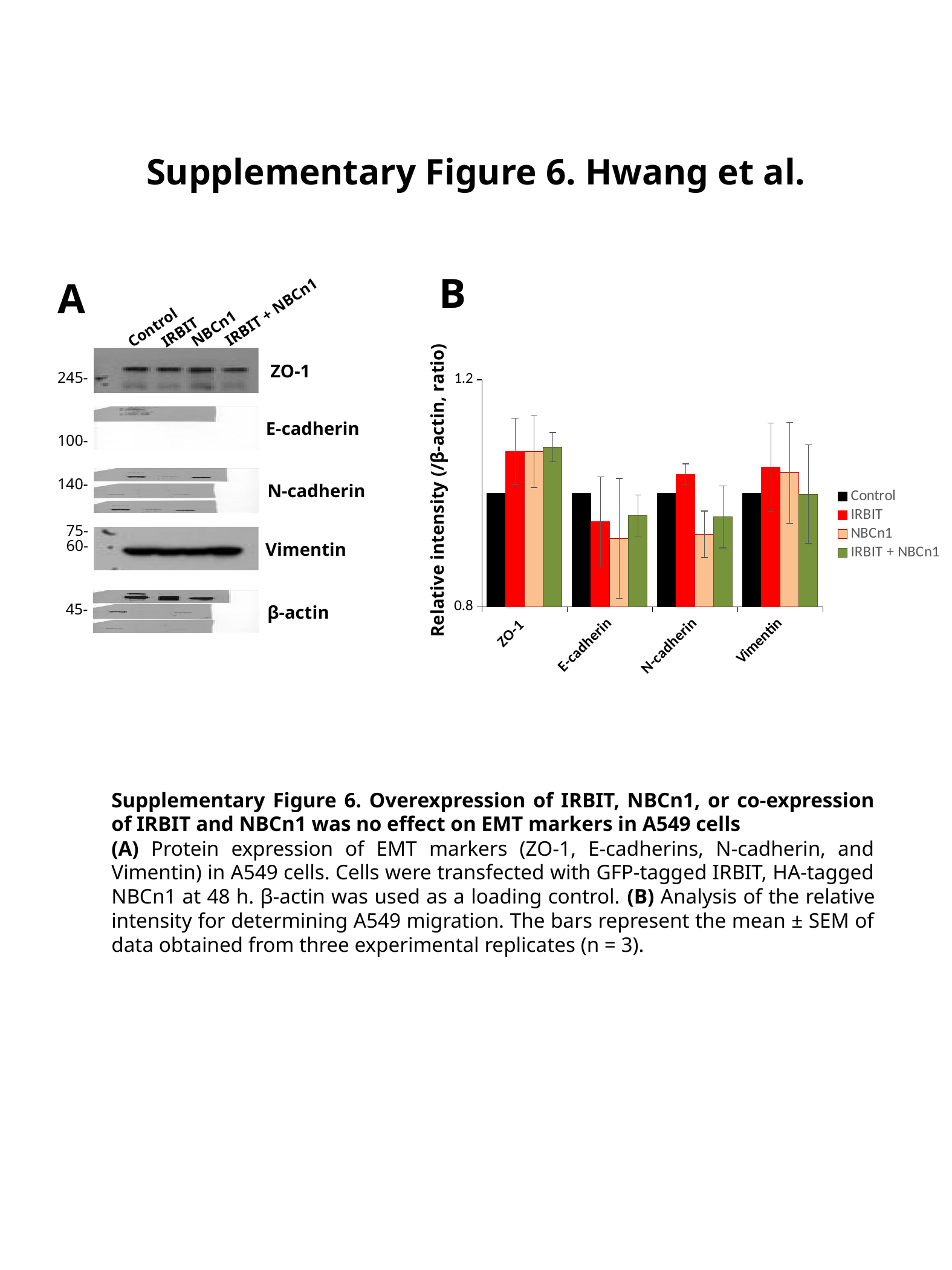

Supplementary Figure 6. Hwang et al.
B
A
IRBIT + NBCn1
Control
NBCn1
IRBIT
ZO-1
245-
### Chart
| Category | Control | IRBIT | NBCn1 | IRBIT + NBCn1 |
|---|---|---|---|---|
| ZO-1 | 1.0 | 1.0737556627856342 | 1.0737412788944964 | 1.081274666425312 |
| E-cadherin | 1.0 | 0.9494980023101953 | 0.9203826222122272 | 0.9605460770327271 |
| N-cadherin | 1.0 | 1.0333620243043466 | 0.9275134757975773 | 0.9580917749505481 |
| Vimentin | 1.0 | 1.0459439612879413 | 1.035780985589863 | 0.9980803140405886 |
E-cadherin
100-
140-
Relative intensity (/β-actin, ratio)
N-cadherin
 75-
 60-
Vimentin
 45-
β-actin
Supplementary Figure 6. Overexpression of IRBIT, NBCn1, or co-expression of IRBIT and NBCn1 was no effect on EMT markers in A549 cells
(A) Protein expression of EMT markers (ZO-1, E-cadherins, N-cadherin, and Vimentin) in A549 cells. Cells were transfected with GFP-tagged IRBIT, HA-tagged NBCn1 at 48 h. β-actin was used as a loading control. (B) Analysis of the relative intensity for determining A549 migration. The bars represent the mean ± SEM of data obtained from three experimental replicates (n = 3).

## Slide 8
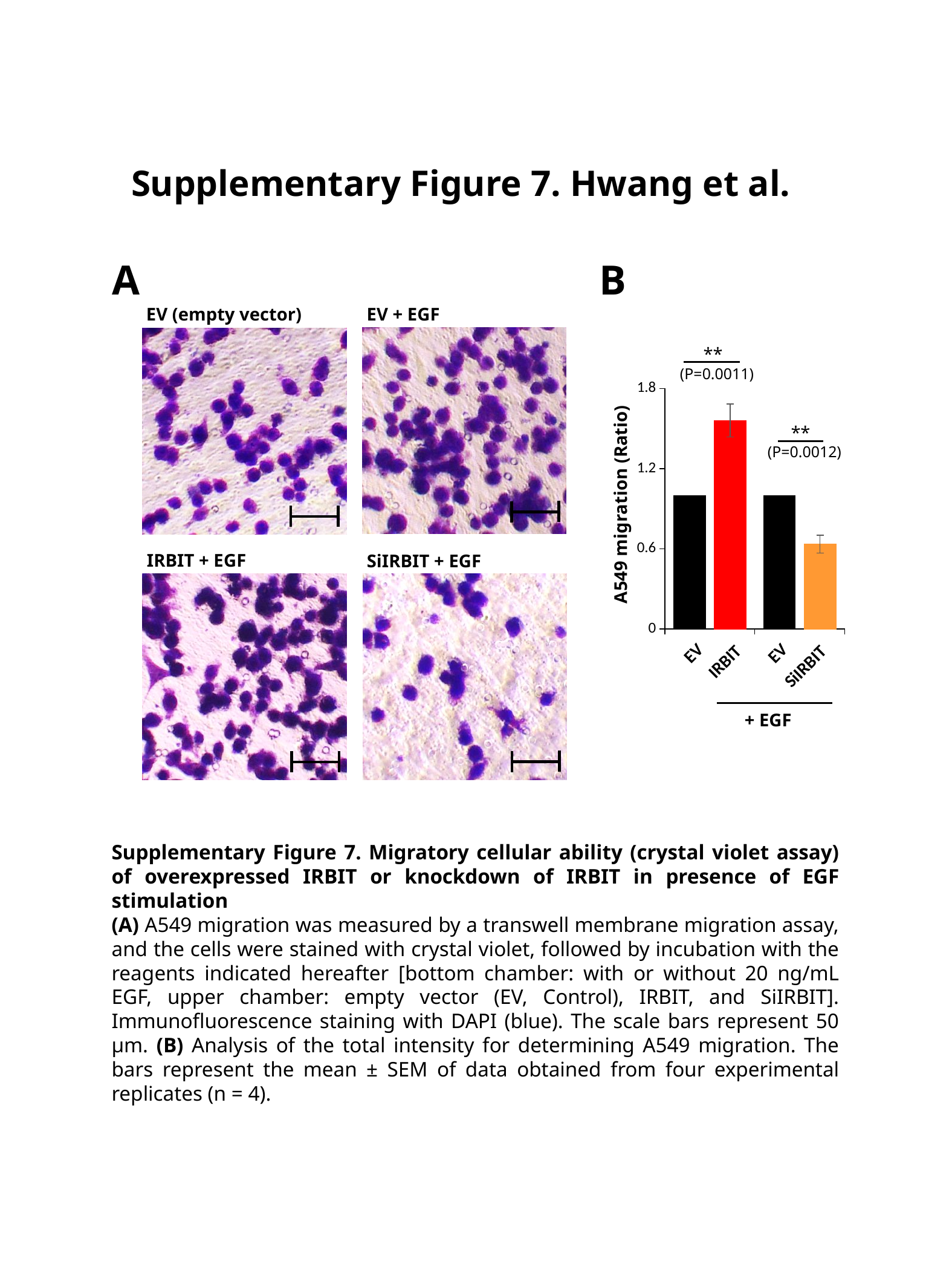

Supplementary Figure 7. Hwang et al.
B
A
EV + EGF
EV (empty vector)
**
 (P=0.0011)
A549 migration (Ratio)
### Chart
| Category | av | |
|---|---|---|
| | 1.0 | 1.5604847030310915 |
| | 1.0 | 0.635471963680273 |**
 (P=0.0012)
IRBIT + EGF
SiIRBIT + EGF
EV
EV
IRBIT
SiIRBIT
+ EGF
Supplementary Figure 7. Migratory cellular ability (crystal violet assay) of overexpressed IRBIT or knockdown of IRBIT in presence of EGF stimulation
(A) A549 migration was measured by a transwell membrane migration assay, and the cells were stained with crystal violet, followed by incubation with the reagents indicated hereafter [bottom chamber: with or without 20 ng/mL EGF, upper chamber: empty vector (EV, Control), IRBIT, and SiIRBIT]. Immunofluorescence staining with DAPI (blue). The scale bars represent 50 μm. (B) Analysis of the total intensity for determining A549 migration. The bars represent the mean ± SEM of data obtained from four experimental replicates (n = 4).

## Slide 9
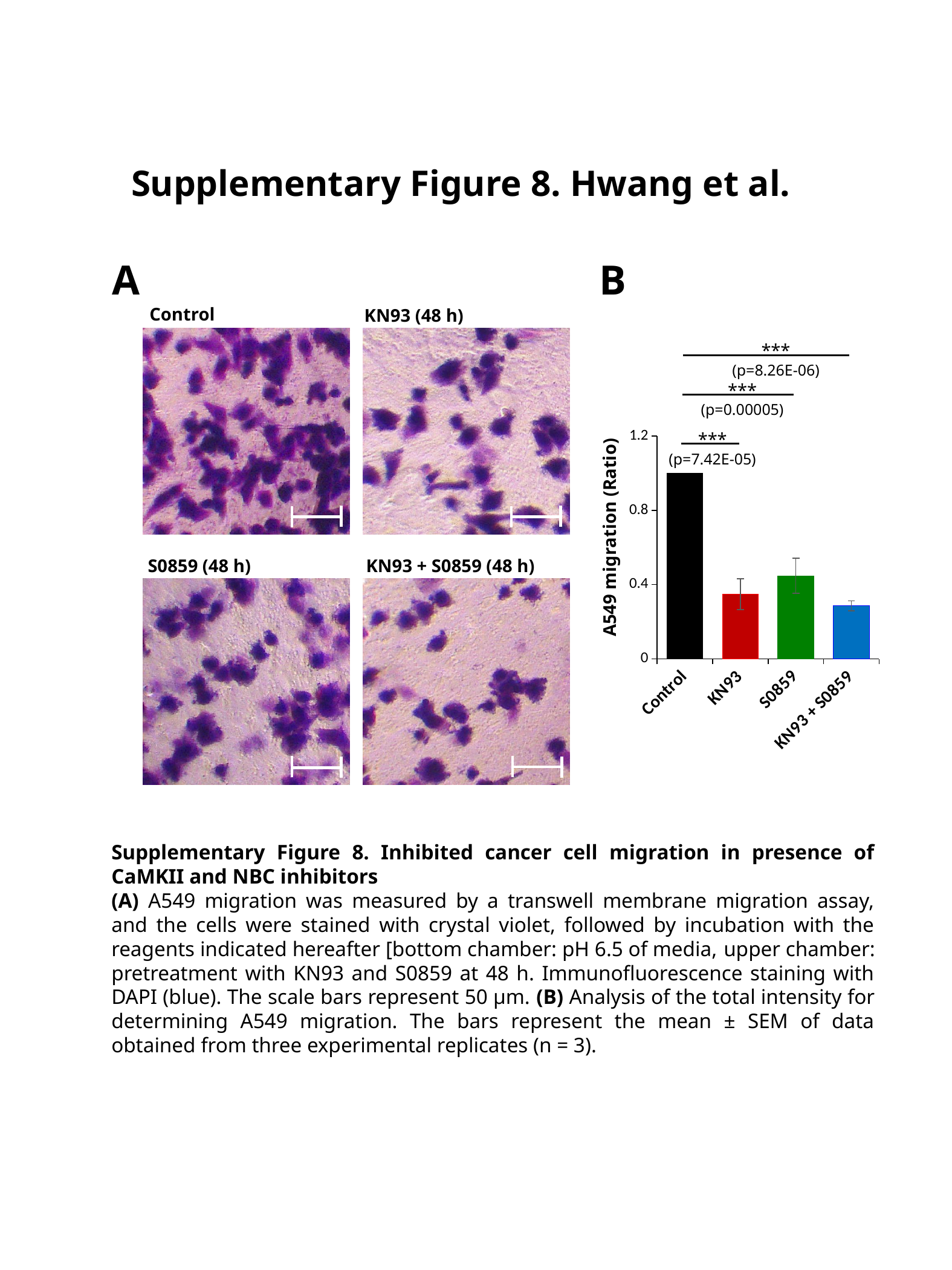

Supplementary Figure 8. Hwang et al.
B
A
Control
KN93 (48 h)
***
(p=8.26E-06)
***
(p=0.00005)
### Chart
| Category | av |
|---|---|
| Control | 1.0 |
| KN93 | 0.34833621859434766 |
| S0859 | 0.447829870004572 |
| KN93 + S0859 | 0.28585048701696447 |***
(p=7.42E-05)
A549 migration (Ratio)
KN93 + S0859 (48 h)
S0859 (48 h)
Supplementary Figure 8. Inhibited cancer cell migration in presence of CaMKII and NBC inhibitors
(A) A549 migration was measured by a transwell membrane migration assay, and the cells were stained with crystal violet, followed by incubation with the reagents indicated hereafter [bottom chamber: pH 6.5 of media, upper chamber: pretreatment with KN93 and S0859 at 48 h. Immunofluorescence staining with DAPI (blue). The scale bars represent 50 μm. (B) Analysis of the total intensity for determining A549 migration. The bars represent the mean ± SEM of data obtained from three experimental replicates (n = 3).

## Slide 10
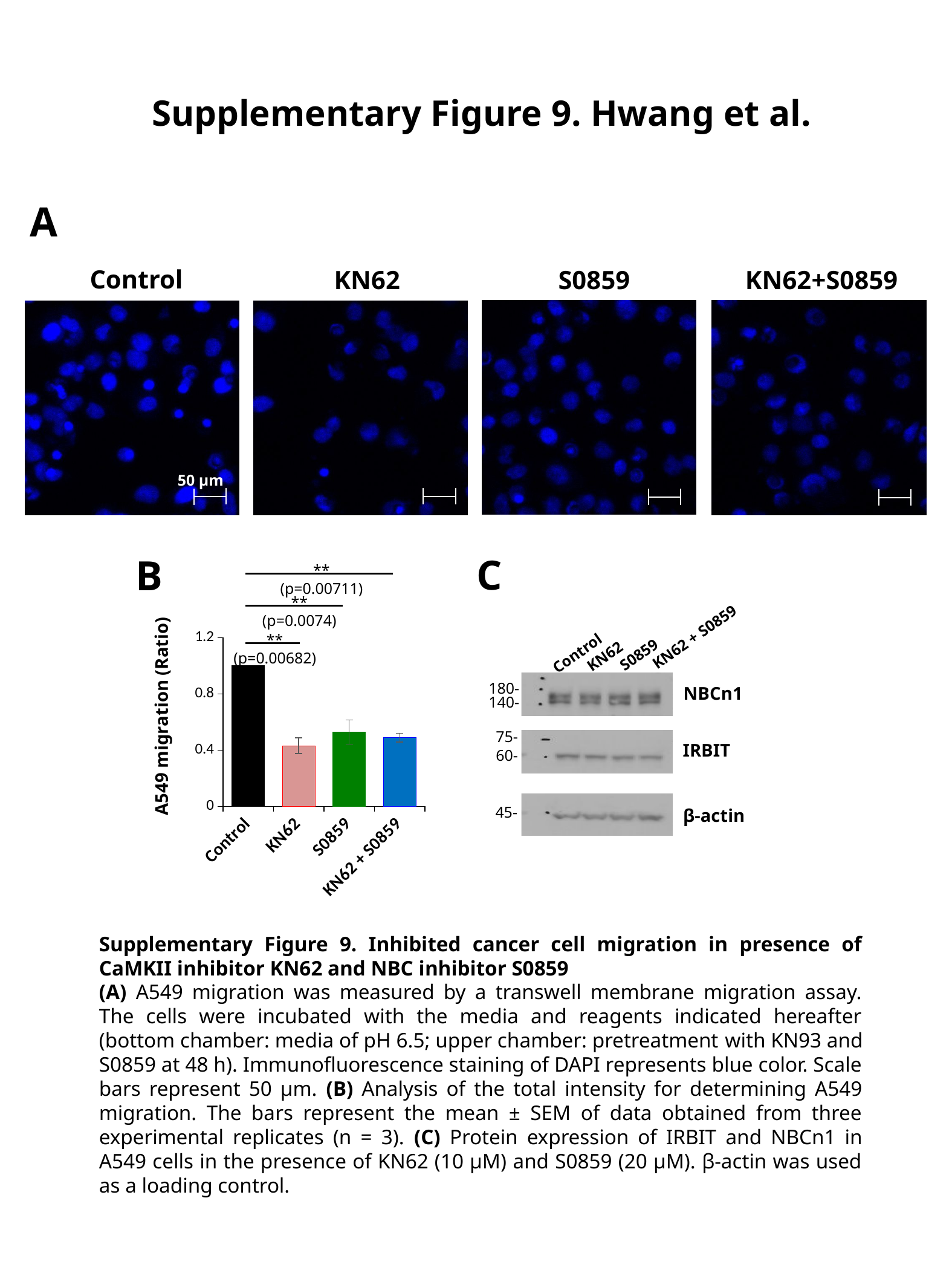

Supplementary Figure 9. Hwang et al.
A
Control
S0859
KN62
KN62+S0859
 50 μm
C
B
**
(p=0.00711)
**
(p=0.0074)
A549 migration (Ratio)
KN62 + S0859
### Chart
| Category | av |
|---|---|
| Control | 1.0 |
| KN62 | 0.43162608698525684 |
| S0859 | 0.5288607012111669 |
| KN62 + S0859 | 0.4882368216548337 |**
(p=0.00682)
Control
S0859
KN62
180-
NBCn1
IRBIT
β-actin
140-
 75-
 60-
 45-
Supplementary Figure 9. Inhibited cancer cell migration in presence of CaMKII inhibitor KN62 and NBC inhibitor S0859
(A) A549 migration was measured by a transwell membrane migration assay. The cells were incubated with the media and reagents indicated hereafter (bottom chamber: media of pH 6.5; upper chamber: pretreatment with KN93 and S0859 at 48 h). Immunofluorescence staining of DAPI represents blue color. Scale bars represent 50 μm. (B) Analysis of the total intensity for determining A549 migration. The bars represent the mean ± SEM of data obtained from three experimental replicates (n = 3). (C) Protein expression of IRBIT and NBCn1 in A549 cells in the presence of KN62 (10 μM) and S0859 (20 μM). β-actin was used as a loading control.
